# Defusing the legal and ethical minefield of epigenetic applications in the military, defense, and security context

**DOI:** 10.1093/jlb/lsad034

**Published:** 2023-12-13

**Authors:** Gratien Dalpé, Katherine Huerne, Charles Dupras, Katherine Cheung, Nicole Palmour, Eva Winkler, Karla Alex, Maxwell Mehlman, John W Holloway, Eline Bunnik, Harald König, Isabelle M Mansuy, Marianne G Rots, Cheryl Erwin, Alexandre Erler, Emanuele Libertini, Yann Joly

**Affiliations:** Faculty of Medicine and Health Sciences, Department of Human Genetics, McGill University, 740 Dr. Penfield Avenue, Montreal, QC H3A 0G1, Canada; Faculty of Medicine and Health Sciences, Department of Human Genetics, McGill University, 740 Dr. Penfield Avenue, Montreal, QC H3A 0G1, Canada; Department of Social and Preventative Medicine, School of Public Health, University of Montreal, 7101 av. du Parc, Montreal, QC H3N 1X9, Canada; Faculty of Medicine and Health Sciences, Department of Human Genetics, McGill University, 740 Dr. Penfield Avenue, Montreal, QC H3A 0G1, Canada; Faculty of Medicine and Health Sciences, Department of Human Genetics, McGill University, 740 Dr. Penfield Avenue, Montreal, QC H3A 0G1, Canada; Medical Faculty Heidelberg, Department of Medical Oncology, Section for Translational Medical Ethics, National Center for Tumor Diseases (NCT), NCT Heidelberg, a partnership between DKFZ and Heidelberg University Hospital, Heidelberg University, Im Neuenheimer Feld, 460, Heidelberg 69120, Germany; Medical Faculty Heidelberg, Department of Medical Oncology, Section for Translational Medical Ethics, National Center for Tumor Diseases (NCT), NCT Heidelberg, a partnership between DKFZ and Heidelberg University Hospital, Heidelberg University, Im Neuenheimer Feld, 460, Heidelberg 69120, Germany; Case Western Reserve University Schools of Law and Medicine, 11075 East Blvd., Cleveland, OH 44106, USA; Human Development and Health, Faculty of Medicine, University of Southampton, Southampton, United Kingdom; Department of Medical Ethics, Philosophy and History of Medicine, Erasmus MC, University Medical Centre Rotterdam, Dr. Molewaterplein 40, 3015 GD Rotterdam, the Netherlands; Karlsruhe Institute of Technology, Institute for Technology Assessment and Systems Analysis (ITAS), PO Box 3640, 76021 Karlsruhe, Germany; Laboratory of Neuroepigenetics, Brain Research Institute of the University Zürich and Institute for Neurosciences of the Swiss Federal Institute of Technology Zürich, Winterthurerstrasse 190, 8057 Zürich, Switzerland; Department of Pathology and Medical Biology, University of Groningen, University Medical Center Groningen, Hanzeplein 1, 9713GZ Groningen, the Netherlands; Department of Medical Education and Psychiatry, Texas Tech University Health Sciences Center School of Medicine, 3601 4th St., Lubbock, TX 79430, USA; Institute of Philosophy of Mind and Cognition, National Yang Ming Chiao Tung University, No. 155, Sec. 2, Linong Street, Taipei 112, Taiwan; Cohen Veterans Bioscience, Data Science, 535 8th Avenue, 12th Floor, New York, NY 10018, USA; Faculty of Medicine and Health Sciences, Department of Human Genetics, McGill University, 740 Dr. Penfield Avenue, Montreal, QC H3A 0G1, Canada

**Keywords:** bioethics, epigenetic clocks and signatures, epigenetic editing, genetic discrimination, military research, child soldiers

## Abstract

Epigenetic research has brought several important technological achievements, including identifying epigenetic clocks and signatures, and developing epigenetic editing. The potential military applications of such technologies we discuss are stratifying soldiers’ health, exposure to trauma using epigenetic testing, information about biological clocks, confirming child soldiers’ minor status using epigenetic clocks, and inducing epigenetic modifications in soldiers. These uses could become a reality. This article presents a comprehensive literature review, and analysis by interdisciplinary experts of the scientific, legal, ethical, and societal issues surrounding epigenetics and the military. Notwithstanding the potential benefit from these applications, our findings indicate that the current lack of scientific validation for epigenetic technologies suggests a careful scientific review and the establishment of a robust governance framework before consideration for use in the military. In this article, we highlight general concerns about the application of epigenetic technologies in the military context, especially discrimination and data privacy issues if soldiers are used as research subjects. We also highlight the potential of epigenetic clocks to support child soldiers’ rights and ethical questions about using epigenetic engineering for soldiers’ enhancement and conclude with considerations for an ethical framework for epigenetic applications in the military, defense, and security contexts.

## I. INTRODUCTION

The concept of the modern-day ‘Super Soldier’ with enhanced physical and psychological resilience could soon become a reality, in part due to scientific advances in the fields of molecular genetics and epigenetics, including the development of techniques to modify (edit) underlying molecular processes. This article is a comprehensive review and analysis of literature, law, and ethics on the potential use of epigenetic technologies in military, defense, and security contexts. The themes discussed in this article derive from a joint, interdisciplinary, workshop of the authors, who are scholars in epigenetics, ethics, and military research that focused on four potential fields of epigenetic applications in the military, defense, and security contexts. The four fields discussed were (a) exposure to nuclear, chemical, or biological weapons; (b) epigenetic age; (c) mental health monitoring; and (d) enhancement of bodily functions.^1^ In these fields, three epigenetic technologies are on the horizon as potential future applications: epigenetic clocks, epigenetic signatures, and epigenetic editing. These are discussed in the present article.

In the first section of the article, we introduce the reader to the scientific background and evidence on epigenetic clocks, epigenetic signatures, and epigenetic editing (see Boxes 1–4), how these technologies and mechanisms might be employed in military contexts, and outline some of the existing regulations and ethical practices in place for epigenetic research in military contexts in different countries (see Box 5). In section two, we formulate scientific, legal, and ethical concerns associated with research on epigenetic clocks and signatures in military contexts related to research data protection, measures to prevent discrimination based on epigenetics, and epigenetic clocks for protecting child soldiers and migrants. In section three, we then discuss four specific ethical and societal issues associated with applications of epigenetic editing in the military, namely, equitable access to potential benefits, the choice of epigenetic editing targets, risks of epigenetic editing, and the potential dual use of epigenetic editing. Finally, in section four, we summarize our ethical and legal considerations and formulate points for a normative framework to defuse the legal and ethical minefield of epigenetics in military, defense, and security contexts.

## II. BACKGROUND

### II.A. Epigenetic Profiling, Signatures, and Biotypes

Epigenetics is the study of biochemical states altering gene activity without modifying underlying DNA sequences. The study of these epigenetic marks of the DNA and/or associated histone proteins, and the changes in genome activity they may entail can provide valuable insight into how life experiences, environmental, and social factors influence genome regulation and contribute to biological functions in health and disease.^2^ However, all biological bases of epigenetics are not yet fully understood by researchers.^3^ Epigenetic profiling examines and analyzes epigenetic marks across an individual’s genome, and has applications which are already surfacing in society, such as informing immigration control, aiding forensic investigations, assessing the approximate age of an individual, and predicting future criminality via the identification of psychopathic neurotype biomarkers.^4^ As the development of epigenetic technology progresses, we are close to seeing fundamental epigenetic research translate into military real-world applications. In 2018, the Defense Advanced Research Projects Agency (DARPA) of the U.S. Department of Defense (DoD) announced a new initiative, the Epigenetic Characterization and Observation (ECHO) program, to develop a man-portable device that enables the detection of an individual’s history of exposure to weapons of mass destruction (WMD), WMD precursors (eg gas, radioactivity), or infectious diseases through profiling of an individual’s epigenome as a ‘footprint’.^5^

Epigenetic technologies are currently being explored for their wide variety of potential benefits within the military context. For example, research is being conducted to identify epigenetic signatures associated with post-traumatic stress disorder (PTSD) in war zone exposed veterans and active-duty soldiers.^6^ In one pilot study, a DNA methylation signature of PTSD (a high severity biotype termed G2) was identified to characterize the biological and clinical heterogeneity of PTSD, along with the development of an improved panel of PTSD diagnostic markers in risk assessment for soldiers, accompanied by the inclusion of a psychotherapy follow-up for a subset of individuals.^7^ However, it will be critically important for the field to establish best scientific practices based on evidence, including independent replication, prospective confirmatory studies, and applicability to non-clinical settings. Any application of such epigenetic signatures associated with ethically sensitive decisions in military and civilian populations should only be conducted after the required high levels of evidence have been accumulated. Applications of this technology are not intended to identify a predisposition to PTSD; rather, their aims are to find out which epigenetic signatures are associated with the presence of PTSD symptoms, which are currently determined by (often ‘subjective’) scales applied in clinical settings. PTSD symptoms can be cognitive, behavioral, and physiological (metabolic, immunological, etc.), thus epigenetic profiling of blood or saliva could provide a reliable readout of such symptoms, more directly than brain-dependent symptoms.^8^ The G2 biotype could conceivably be used as an alternative method to monitor the effects of psychotherapy in soldiers suffering from PTSD.^9^ There is an additional opportunity to utilize the history of soldiers’ epigenetic data to demonstrate that their disability was acquired in the context of work. By using epigenetic signatures such as the G2 biotype, the military could follow soldiers’ exposure to chemical weapons or trauma (eg PTSD symptoms) after troop deployment in combat zones and use this information as evidence of disability caused through duty. To this end, DARPA’s portable footprint device represents a potential future application of epigenetics that could benefit soldiers.

### II.B. Epigenetic Clocks

In another pilot study, the epigenetic testing technology called ‘GrimAge’, an epigenetic biological clock, is used to reveal the biological age of a person, in contrast to their chronological age, allowing for more accurate lifespan predictions.^10^ Higher DNA methylation identified by GrimAge is associated with lower cognitive ability and a higher likelihood of vascular brain lesions as individuals age, regardless of their early-life cognitive ability.^11^ In other words, these epigenetic signatures mark different measures of brain health to aid in the prediction of age-related cognitive decline. There is a potential interest in eventually using GrimAge to provide soldiers with epigenetic information about their risk for cognitive decline as information to consider when deciding whether they would still want to go for high-risk missions or seek their approval to use such information to help select soldiers for high-risk missions.^12^ Furthermore, extensive research is aimed at assessing biological clocks’ potential for quantifying biological aging rates, testing longevity, and for rejuvenating interventions.^13^ However, knowledge gaps remain in understanding epigenetic clock mechanisms and biomarker utilities, such as the drivers and regulators of age-related changes in single-cell, tissue- and disease-specific models. There is a need to further investigate other epigenetic marks and conduct longitudinal and diverse population studies in humans and non-human models. These challenges currently limit the accuracy of GrimAge in predicting the trajectory of biological aging in individuals. For instance, even if the associations between markers of biological aging and aging phenotypes are significant, the effect sizes tend to be small.^14^

Epigenetic age clocks could also be used to determine the (chronological) age of presumed child soldiers in conflict zones to protect their rights, health, and well-being.^15^ Many of the available anatomical tests to help determine the age of individuals lacking identity documents are often cumbersome, invasive, and unreliable. Thus, countries within proximity of conflict zones might be tempted to use epigenetic age clocks to primarily predict, estimate, or corroborate chronological age to supplement additional biological tests and facilitate their decision making about child soldier claims. However, the results of epigenetic clocks for children deviate between populations. The poor test performance and variability of epigenetic age clocks in children need to be addressed before such clocks are implemented.^16^

Importantly, epigenetic clock technologies (ie to estimate biological or chronological age) need to be validated in the respective target population (eg longitudinal studies, ethnicity, or population studies) before actionable uses for epigenetic clocks can be proposed for vulnerable populations such as child soldiers.^17^

### II.C. Epigenetic Editing

Finally, recent advances in site-specific epigenetic editing technologies, if ever validated for use in humans, could be employed in the future to attempt to make soldiers psychologically more aware, more resilient, or adaptive in active combat contexts.^18^ The formation of memory is a naturally occurring example of epigenetic editing requiring extensive cellular and molecular changes in brain regions. It has been established that epigenetic mechanisms, and DNA methylation in particular, maintain cellular identity over successive cycles of cell division by self-perpetuating mechanisms and may be a mechanism to help store information in memory over time.^19^ Studies in mice have indicated the role of epigenetic mechanisms in the consolidation of fear memories and the development of PTSD, which would be of interest to the military.^20^ The interest of epigenetic editing for soldiers could also be, speculatively, when delivered in the periphery or a specific organ, to provide a better resistance to toxic chemicals. Moreover, it may help reverse deleterious health states impacting performance such as potentially acquired or inherited PTSD-predisposing epigenetic signatures. However, the use of this technology in humans commands caution: (1) the clinical application of epigenetic editing has not yet started and is not yet ripe; (2) the complexity and ubiquity of epigenetic changes make it difficult to identify those induced by specific social, economic, or contextual factors; (3) the causality between epigenetic changes and symptoms, especially for complex functions like cognition or memory, is not yet proven; (4) more thorough investigations of the ELSI (ethical, legal, and social issues) of the potential use of human epigenetic editing in general, but particularly in the military as well as the civilian context, are needed.^21^ The current insufficient research and the need to demonstrate the long-term efficacy of epigenetic editing in mammals makes the use of this technology for any human applications still premature.^22^ However, it is expected that further preclinical therapeutic successes will be achieved and heading to the initiation of clinical trials, as indicated by the development of various companies dedicated to exploit epigenetic editing to treat diseases.^23^

### II.D. Boxes 1, 2, 3, 4: Scientific Summary on Epigenetics, Epigenetic Editing, Epigenetic Profiling, and Multigenerational Epigenetic Effects

**Box 1: Definition of Epigenetics**

Epigenetics is the study of the biochemical states altering genome activity that do not change the underlying DNA sequence. These biochemical modifications can influence higher-order DNA structures (eg chromatin), which regulate the function, structure, and integrity of genetic material.^24^ Although all cells within a given organism share a similar DNA sequence with all of the same genes in each cell, differential gene expression during development is responsible for the many different cell types, tissues, and organs and is an example of epigenetic regulation.^25^ An additional example of epigenetics is learning. When learning something, new epigenetic marks are formed in neuronal tissue via histone acetylation and DNA methylation, both of which can influence each other during learning.^26^ The loss of coordination of epigenetic machinery and marks with age has been proposed to underlie cognitive impairment.^27^

Post-translational modifications on histone tails or core, or direct nucleotide modifications on DNA such as methylation, are epigenetic marks that can impact genome activity and gene expression on a large scale.^28^

**Box 2: Epigenetic Editing**

Epigenetic interfering technologies can be divided into two broad types: genome-wide pharmacological interventions that non-specifically modify the epigenome (inhibitors of epigenetic writers, erasers, and readers) and site-specific epigenetic editing. The former uses drugs such as histone deacetylase inhibitors or DNA methyltransferase inhibitors to confer the same epigenetic change throughout the entire epigenome. The latter uses genomic engineering technologies to confer epigenetic changes only at specific epigenome targets and possibly in a given cell type.^29^ Epigenetic editing entails inducing or removing an epigenetic state at a particular genomic locus at will. This can modulate the activity or expression of a target coding sequence or a regulatory element that controls one or several gene(s).^30^

Recently developed tools for site-specific epigenetic editing are variants of the CRISPR-Cas9 system, which use a nuclease-deactivated dCas9 module fused to an epigenetic modifier of choice. The CRISPR-dCas9 module loaded with a single guide RNA (sgRNA) is used to locate the fused epigenetic modifier to a specific region on the genome, thus ensuring that the expected epigenetic change is only induced by the modifier at a desirable location. CRISPR-dCas9 fused to modifiers can function as erasers (eg a deoxygenase enzyme like TET1 that can promote DNA demethylation thus potentially activating gene expression) or writers (eg a methyltransferase like DNMT3A that can repress gene expression) of targeted gene loci.^31^ As with any genome editing technology, although significant improvements have been achieved, both toward sustained upregulation as well as maintained repression by using combinations of epigenetic effector domains, there are still important limitations to epigenetic editing.^32^ Indeed, such limitations in humans include maintaining the long-term stability and persistence of modified epigenetics signatures over time and mitigating the presence of off-target effects while boosting the signal of on-target edits though the persistence of methylated CGP islands has been demonstrated in mammals.^33^

**Box 3: Epigenetic Profiling**

As epigenetic factors and mechanisms are essential for biological processes and their regulation, they are key targets for efforts aiming at profiling individuals’ epigenome.^34^ Epigenetic profiling consists of capturing ‘snapshots’ of the state of one or several epigenetic marks at a given time and in specific tissues. Such marks can include the methylation status of DNA strands, histone modifications (eg acetylation, methylation), or chromatin structure in correlation to genome activity. Examples of fine-tuned epigenetic profiling are ‘epigenetic clocks’, which use the level of DNA methylation on specific genomic loci in blood or saliva to determine a person’s relative health or chronological age. These epigenetic profiles may be useful in studying the progression of biological aging and of diseases, for disease diagnosis, and may also act as markers for evaluating therapeutic interventions such as psychotherapy for PTSD.

**Box 4: Multigenerational Epigenetic Effects**

The possibility of identifying epigenetic signatures associated with diseases and trauma or to recreate persistent and stable epigenetic signatures in humans also entails considering the consequences of epigenetic inheritance for future generations. Epigenetic inheritance implies that epigenetic changes induced by exposure are present in the reproductive cell (the gamete, which is the oocyte or sperm cell) that generates the offspring and are passed on to that offspring. If epigenetic changes are present only in somatic cells (all cells except germ cells), they will not be inherited by sexual reproduction. While preliminary research suggests that certain epigenetic marks can be passed on to subsequent generations (intergenerationally and transgenerationally) in experimental animals, evidence of epigenetic inheritance in humans is virtually nonexistent and almost impossible to provide, in part because of confounding factors and logistical, financial, and ethical hurdles that limit epidemiologic studies spanning multiple generations.^35^

Of particular concern, given current research findings, is the possibility of epigenetic inheritance of trauma exposures (eg PTSD) through epigenetic mechanisms, which encompasses significant environmental experiences that can modify a person’s behavior, cognition, metabolism, and physiology.^36^ Emerging evidence in animal studies suggests the possibility of inheritance of traumatic events through epigenetic mechanisms.^37^

### II.E. Research Ethics Guidelines and Regulations

Military life requires a high level of psychological strength and physical fitness. Army personnel can find themselves in extreme circumstances; for instance, they may need to participate in missions in a foreign territory for prolonged periods. In these circumstances, officers and military scientists could have an interest in using genetic and epigenetic information for selecting the fittest personnel for the troops. Within this context, epigenetic testing might provide additional options to those offered by genetic tests, to further tailor diagnostics, treatments, enhancements, and assess the future risk of developing a medical condition. As in the civilian context, this enterprise should abide by the ethical principles developed to guide biomedical research involving human subjects, notably those outlined in the *Belmont Report*, namely, non-maleficence and beneficence, justice, and respect for persons and those outlined in international ethical guidelines including the *UNESCO Universal Declaration on Bioethics and Human Rights*, the *WMA Declaration of Helsinki—Ethical Principles for Medical Research Involving Human Subjects*, and the *CIOMS International Ethical Guidelines for Health-related Research involving Humans*.^38^

However, the application of the principle of informed consent based on the conception of individual autonomy can be hindered in the military context by informal codes of compliance and specific morale established within the military culture. Key values upheld within the military include selflessness, the duty to follow orders, accountability, and the obligation to look out for the welfare of one’s subordinates.^39^ These values reflect the importance of respect of autonomous decisions, beneficence, and non-maleficence, but additionally also the need for considering respect for authority and self-sacrifice. The latter can interfere with the application of biomedical ethics principles (based on autonomy and beneficence) in military research.^40^ For example, in a culture where it is generally difficult, or considered ill-advised, for lower-rank individuals to voice their dissent, the ability to freely consent to medical research can be compromised. Also, as in civilian research ethics, incentives such as the promise or expectation of promotions in rank could influence soldiers to accept dangerous missions after undergoing biological enhancements and compromise their capacity to provide free and informed consent.^41^ However, as some of these tensions and controversies may be unavoidable, various jurisdictions have implemented policies to try to safeguard soldiers’ autonomy and well-being when participating in biomedical research.

### II.F. Box 5: Comparative Summary of Canada, USA, United Kingdom, and European Union Research Policies for the Safeguard of Soldiers’ Autonomy and Well-being

**Canada**

- Research involving human participants (including soldiers) that is conducted under the auspices of institutions eligible for public funding by the three main federal research agencies must comply with the Tri-Council Policy Statement: Ethical Conduct for Research Involving Humans (TCPS2), a joint research ethics policy. Yet, the compliance of governmental agencies conducting research to TCPS2 rules is voluntary.^42^ The Department of National Defence of Canada has agreed to adhere to TCPS2.^43^
- When addressing the voluntariness of consent for members of the Canadian Armed Forces (CAF), TCPS2 provides warnings about undue influence from commanding officers.
- To maintain the voluntariness of research participants as much as possible, TCPS2 emphasizes the importance of not using incentives to enroll, and allowing participants to withdraw at any time, thus putting an end to all data and sample collection.^44^
- Moreover, the policy document Research Involving Human Subjects from the Department of National Defence also states that all CAF members are required to provide free informed consent and retain the right to withdraw without any form of constraint or coercion.^45^

**United States**

- US soldiers participating in epigenetic research in military settings are protected by the Department of Defense (DoD) research policy, which includes the principles of respect for the person, beneficence, non-maleficence, and justice reflected in the *Common Rule* and adapted to the military context.^46^
- No soldier can be compelled to consent to participate in defense-funded or conducted medical research.^47^
- When defining the minimal risk of a research project, DoD policies should only take into consideration the inherent occupational risks that certain participants face in their everyday life, not those met by soldiers while on duty (see subsequent section on risk-assessment).^48^
- The involvement of soldiers in research involving risk deemed greater than minimal requires an institutional research board (IRB) approval and the appointment of an ombudsperson who will ensure the presence of safeguards for research participants likely to be vulnerable to coercion or undue influence.
- These safeguards are
  – IRB-approved information including that which can be facilitated by digitally provided material;
  – Prohibition of compensations for participation in research;
  – Mandatory presence of the ombudsperson during informed consent sessions;
  – Absence of conflicts of interest of the ombudsperson.^49^

**United Kingdom**

- In the UK, the research guidance document Governance of Research Involving Human Participants from the Ministry of Defense states that in assessing the suitability of a research protocol, the Ministry of Defense Ethics Committee (MODEC) must consider the vulnerability of participants, in particular when obtaining informed consent for the recruitment of junior soldiers.^50^
- The MODEC also assumes a general role in preserving and protecting the dignity, rights, safety, and well-being of research participants.
- These measures were prompted by past abuse of human participants in experimental military research.^51^

**European Union**

- A 2014 regulation issued by the European Parliament and the Council of the European Union, applying to all its member states, requires that no undue influence should be ‘exerted on participants to participate in the clinical trial’.^52^
- In a similar manner, the *Additional Protocol to the Convention on Human Rights and Biomedicine Concerning Biomedical Research* states that ethics committees should be satisfied that no undue influence, including a financial one, should be exerted on persons to participate, with special attention paid to vulnerable persons.^53^
- Within this context, vulnerable persons are defined as including persons fulfilling their military service.^54^

### II.G. Risk–Benefit Assessment

Considerations pertaining to ethical decision-making in medical research can differ between military and civilian contexts. An example where such difference is manifest is risk–benefit analysis. In civilian bioethics, medical research on human subjects is generally deemed inappropriate if risks to the research subject greatly outweigh expected benefits obtained for the individual, a group presenting with the same condition, or the human population at large. In TCPS2 (Canada) for instance, minimal risk in (civilian) research is defined as research in which the probability and magnitude of possible harms implied by participation in the research is no greater than those encountered by participants in the different aspects of their everyday life.^55^ Like in civilian research where more than minimal risk can be tolerated if the potential individual or group benefits are high or the alternatives are dismal (eg last line oncological patients), risk–benefit assessments in the military context may also consider the broader norms of the military culture, where judgments about risk are made in consideration of the military advantage to be gained: greater risks to individual research participants may be weighed against important military gains.^56^ Because such decisions may not meet civilian research ethics standards, policy requiring specific ethics approval for research involving more than minimal risk to participants (eg see above U.S. DoD policy) might contribute to maintaining the safety of research protocols in the military context.^57^

Another point to consider, particularly relevant to epigenetics, is that the degree of health risk may depend on whether the result of a medical intervention is permanent, long-term, temporary, or reversible.^58^ For instance, epigenetic editing offers the possibility to alter gene expression without modifying the genome sequence. Compared to DNA sequence alterations, such modifications are more likely to be reversible and can theoretically be accomplished by lifestyle changes, drugs, and targeted epigenetic editing technology (eg CRISPR-dCas9 fused to writers and erasers of epigenetic marks, see Box 2).^59^ However, adverse effects from an epigenetic intervention may eventually or inadvertently impact military and non-military life.^60^ For instance, there is a major concern about the potential for modifications to germ cells being passed on to future generations, thus causing changes to a soldier’s offspring.^61^ The possibility that a targeted epigenetic modification can later be reversed to its original state conceptually reduces the risk of potential long-term adverse effects and of passing on such modification to the next generation.^62^ A prerequisite of this assumption is robust clinical data ascertaining if targeted interventions (eg with CRISPR-dCas9 technology) only modifies somatic cells’ epigenome, the possibility of reversibility and a plan for such reversal in the research protocol, and robust data that such reversal occurs safely. Such data have not yet been validated. However, one has to keep in mind that targeted epigenetic editing research is still at a very early stage.

## III. RESEARCH ON EPIGENETIC CLOCKS AND SIGNATURES IN MILITARY CONTEXTS: ETHICAL, LEGAL, AND SCIENTIFIC CONCERNS

Since the genomic revolution of the late twentieth century, greater attention has been given to categorizing, correlating, and explaining health states based on individual genetic differences. Epigenetics is no exception to this narrative, as it very much supports the hypothesis of individual differences at a molecular level between humans correlating with health states. Here, in an anticipatory and prospective manner, we highlight some ethical and policy concerns with epigenetics research on humans based on hypothetical scenarios derived from the context of the chain of command culture within the military. Our aim is to prompt military officers, researchers, and other stakeholders to reflect on the opportunities and challenges of epigenetics research and technology. However, the overarching goals of taking better care of soldiers’ health should be kept in consideration, as well as improving their general performance when facing physical and psychologically demanding circumstances.

### III.A. Ethical Evaluation of Research on Epigenetic Clocks and Signatures in the Military: Military Use of Epigenetic Research Data

Adding to the ethical challenges accompanying the generation, use, and storage of epigenomic research data in the general population, such as privacy issues, there is a concern that epigenetic data collected from soldiers could eventually be used to discriminate against them.^63^ There is a parallel to be drawn with the use of soldiers’ genetic data. The military may decide to screen enlistees and service members for certain genomic variants associated with undesirable traits with the goal to exclude individuals for service assignments. For example, those with increased risk of injuries in the battlefield may be perceived as vulnerable for certain combat missions.^64^

Using epigenetic research as a means of biological profiling can perpetuate the erroneous perception that a person’s epigenetic profile is deterministic of their physiological and psychological characteristics. This view of epigenetics can lead to institutionalized forms of discrimination within the military hierarchy. In this scenario, high-ranking officers could use data in epigenetic databases to select, promote, or terminate contracts of employees based on epigenetic signatures. For example, epigenetic biological clock signatures indicative of premature aging, or with an estimated biological age that greatly surpasses chronological age, a trend commonly seen in trauma survivors, or associated with a lack of resilience to trauma induced by combat, could be used for this purpose.^65^

If profiling based on epigenetic data and models does not accurately demonstrate features such as a low resilience to trauma in a particular individual, its substitution of a soldier’s actual performance and merit may lead to an unfair selection of military employees, which may then hinder future opportunities for growth or promotion of deserving candidates. If evidence of the predictive accuracy of epigenetic profiles is lacking, their use may be harmful not only to individual candidates, but also to the success of future military operations. However, if epigenetic tests eventually achieve high predictive power, officers might be required to use them to protect soldiers’ health and safety (through selection of soldiers for dangerous missions). Considering the military employees’ rights (as research participants) not to be informed about their epigenetic test results if they wish, the release of epigenetic data to research participants themselves or to their superiors might be deemed unethical. Since epigenetic data is sensitive information, safeguards must be put in place by analogy to handing out genetic and genomic research data.^66^

### III.B. Legal Evaluation of Epigenetic Research on Clocks and Signatures in the Military: Are Frameworks to Prevent Discrimination Fit for Purpose?

Worldwide, several countries have developed genetic non-discrimination policies.^67^ However, to our knowledge, none of them, including the *International Declaration on Human Genetic Data*, explicitly addresses discrimination based on epigenetic information.^68^ We compare here the potential effect of genetic non-discrimination policies from Canada and the USA to protect soldiers’ epigenetic information.

Designed to prevent genetic discrimination, the *Genetic Non-Discrimination Act* (GNDA) is a Canadian federal law prohibiting to compel an individual to undergo genetic testing or share the results of their genetic tests for goods and services agreements or entering contracts, including in the context of insurance or employment. The GNDA amends the *Canada Labour Code* (CLC) to include similar provisions protecting against the use of genetic test results in the context of employment in the federal government.^69^ The GNDA also amends the *Canadian Human Rights Act* (CHRA) to include ‘genetic characteristics’ as an illicit ground of discrimination.^70^ As specified in the *National Defence Act*, members of the Canadian Forces are subjected to any acts of the Parliament of Canada, including the GNDA.^71^

To clarify the scope of protection offered, the GNDA defines genetic tests as the analysis of DNA, RNA, or chromosomes for purposes such as the prediction of disease or vertical transmission of risks, or monitoring, diagnosis, or prognosis.^72^ However, the question remains as to whether the language of this definition can be broadly interpreted to cover the use of epigenetic test results of soldiers.^73^ When trying to determine if personal epigenetic information of soldiers is covered under the GNDA, we note that both the G2 biotype and GrimAge, two epigenetic signatures cited as examples above, make use of methylated DNA signatures. This can conceptually be considered as a DNA analysis that allows for the monitoring of health status or for the diagnosis of conditions in soldiers who have potentially been exposed to warfare and/or chemical weapons. Moreover, animal studies show the possibility for PTSD-related epigenetic signatures to be transmitted to subsequent generations if present in germ cells, thus aligning with the general understanding of the vertical transmission of risk mentioned in the GNDA definition of genetic tests.^74^ Therefore, in Canada, there is a possibility that the GNDA provides a legal protection against the discriminatory use of soldiers’ epigenetic test results. The limitations and extent of that protection provided, however, are unclear given the genetic centered definition of what is prohibited. In the obiter dictum (a non-legally binding opinion) of the 2020 decision by the Supreme Court of Canada bearing on the constitutionality of the GNDA, the court suggested that the scope of ‘genetic characteristics’ is not fixed in time and may allow for a broader range of genetic information than the molecularly formulated definition of genetic tests applying to the context of entering or continuing a contract or an agreement and the provision of goods and services.^75^ Following this interpretation by the court, we suggest that the term ‘genetic characteristics’ could be broadly interpreted in the future to include epigenetic information, whether acquired directly by exposure (eg epigenetic signatures of PTSD) or inherited from an exposed parent (eg inheritance of epigenetic signatures of PTSD from her/his exposed parent via gametes).

The *Genetic Information Non-Discrimination Act of 2008* (GINA) was enacted in the USA to protect individuals against discrimination based on their personal genetic information, as it applies to health insurance and employment. These protections are intended to encourage Americans to take advantage of genetic testing as part of their medical care. It also has the effect of replacing the more narrowly formulated EO13145 intended to prohibit genetic discrimination in Federal Employment.^76^ However, GINA does not cover soldiers because the laws amended by GINA do not apply to them.^77^ Even if soldiers were protected under GINA, the current definition of genetic tests remains unclear about the protection it confers to epigenetic information.^78^ Although not required by law, the US military has adopted as part of its *Equal Opportunity Directives*, a policy prohibiting unlawful genetic discrimination.^79^ It is unclear what actions the policy prohibits since the term ‘unlawful discrimination’ is not defined therein. For greater consistency, these terms could be interpreted to prohibit genetic discrimination in a similar manner to the framework proposed by GINA, but as stated, it is not clear to what extent GINA would protect epigenetic information.

The uncertain application of traditional genetic non-discrimination laws to epigenetic information derived from soldiers participating in epigenetic research is cause for concern. This concern originates from the limited scope and definitions of older narrowly formulated genetic non-discrimination policies that may not extend coverage to epigenetic tests or edits. Furthermore, soldiers are not necessarily covered under general genetic non-discrimination protections (eg GINA in the USA).

### III.C. Epigenetic Clocks for Protecting Child Soldiers and Migrant Populations—Scientific, Legal, and Ethical Concerns

DNA methylation clocks that measure the age or the aging process of a person are another emerging application of epigenetics that may potentially hold benefits for the military and in the context of national security. A prevalent issue in the conduct of war nowadays is the use of child soldiers. As defined by the *Paris Principles and Guidelines on Children Associated with Armed Forces or Armed Groups*, a child soldier is ‘any person below 18 years of age who is, or who has been, recruited or used by an armed force or armed group in any capacity’.^80^ Given the severe implications for their emotional and physical well-being, several conventions prohibit the use of child soldiers in armed conflict: Articles 1 and 2 of the *Optional Protocol to the Convention on the Rights of the Child on the Involvement of Children in Armed Conflict* stipulate that State Parties should ensure that children under the age of 18 shall not ‘take direct part in hostilities’ and shall not be ‘compulsorily recruited into their armed forces’, and the *Rome Statute of the International Criminal Court* (Rome Statute) establishes that ‘conscripting or enlisting children under the age of fifteen years into the national armed forces or using them to participate actively in hostilities’ is a war crime.^81^ As child soldiers are always considered victims of war, regardless of their role, states have a vested interest in their identification and in ensuring that they are not being involved in combat. Moreover, child combatants under the age of 15 are afforded privileged treatment, in that even when taking direct part in combat, they continue to benefit from the legal protection provided by the *Protocol Additional I and II to the Geneva Conventions*.^82^

Within this context, a potentially positive application of DNA methylation clocks is to determine the age of a person (ie chronological age), which can be used to identify children whose status as child soldiers is otherwise impossible to confirm via traditional identity papers.^83^ Countries that have ratified the *Rome Statute* or the *Optional Protocol* might have strong interests in the use of these clocks to identify potential child soldiers in combat, or to verify claims of those seeking the status of child soldiers due to the legal protection afforded to them. Current methods of age verification, besides legal documents, such as examining X-ray images of certain bones (eg left hand and wrists, clavicles, and knees) and examining teeth, are considered time-consuming and imprecise with wide margins of error.^84^ Epigenetic age clocks conducted with a buccal swab could offer a way to screen child soldiers, as an alternative to skeletal maturity estimates, eg by X-rays.^85^ However, given that an individual is only considered a child soldier if she/he is below the age of 18, it is uncertain as to whether epigenetic clocks will be able to accurately differentiate an individual of 15 years of age and one of 18. Currently, epigenetic age clocks are only able to roughly estimate age, with reported median errors between 2.7 and 3.6 years.^86^ This lack of accuracy highlights the scientific and ethical challenges presented by epigenetic age clocks, and their potential life-changing consequences. If epigenetic age clocks are the only method used to verify age in the absence of identification papers, there is the potential for minors aged 15–17 to be classified as adults, and thus be excluded from benefiting from the legal protections that they would otherwise be afforded as child soldiers. Inaccuracies of epigenetic age clocks for individuals under 20 years old is well known.^87^ However, a recent report of a Pediatric-Buccal-Epigenetic clock based on individuals aged between 0 and 19.5 years presents an improved median error of 0.35 years, showing that epigenetic age clocks with a better accuracy might become a benefit to estimate minors’ age in coming years.^88^

Furthermore, epigenetic biological clocks that can detect and capture the effects of trauma and stress (eg GrimAge) can be useful to help document the damages, in particular the long-term psychological consequences (eg PTSD symptoms), that result from a child’s involvement in armed conflict (eg malnutrition, lack of adequate living conditions, psychological distress).^89^ Epigenetic biological clocks could serve as quantitative evidence of the adversity undergone, with the potential to inform government response and policy in aid for child soldiers, and their reintegration into civil society. If validated with appropriate reference groups, epigenetic biological clocks could improve screening for risk stratification and diagnosis, which could promote early intervention to prevent and treat PTSD and other consequences of war and violence for these child soldiers.^90^

A potential problem with epigenetic age clocks based on DNA methylation devised to estimate chronological age is that they can also capture the ‘biological’ aging processes associated with exposures to environmental features, which are usually reflected in epigenetic biological clocks.^91^ The unresolved question is how these epigenetic clock estimations of the chronological age and of biological aging acceleration can be distinguished in the context of child soldiers exposed to the trauma of war, persecution, and torture. Child soldiers might, in the eye of an epigenetic age clock, appear as being older than their actual chronological age, due to exposure to trauma (ie typically captured by epigenetic biological clock), and thus be misclassified. The ‘calibration’ of each type of clock^92^ (chronological age vs biological age) against large sample size populations exposed to different traumas and from different ethnic groups should help improve the precision and specificity of epigenetic clocks.^93^

Notwithstanding its potential benefits, testing the use of epigenetic biological and age clocks applied to child soldiers, which requires epigenetic research first, also presents significant ethical challenges. Obtaining informed consent from these children can be challenging as they may not have sufficient intellectual maturity and/or legal capacity to provide consent and are potentially impacted by communication barriers, cultural differences, poor health, and limited scientific literacy. Moreover, their vulnerable status due to the displacement, trauma, and social situation they are experiencing makes it difficult to consider their consent as ‘freely given’ unless robust measures are put in place to avoid coercion and undue pressure, perceived or real.^94^

## IV. EPIGENETIC EDITING IN THE MILITARY AND ASSOCIATED ETHICAL AND SOCIETAL CONCERNS

### IV.A. Equitable Access to Potentially Beneficial Interventions

When soldiers take part in a military operation, they put their physical and mental well-being on the line. Any enhancements could thus serve as additional protection from potentially harmful long-term side effects, alongside the standard advantages provided to them through training, weapons, and armor.^95^ As described in the previous section, genomic science can improve a unit’s effectiveness by selection/exclusion of individuals based on genetic signatures predicting and how they are expected to function in different environments. In addition, gene-based modifications can further be used to improve warfighting abilities of individual soldiers.^96^ As suggested in a 2014 report, soldiers could be genetically engineered to need less sleep, thus enabling them to carry out missions despite lack of sleep.^97^ Conceptually, there is also the possibility of using epigenetic-based technologies, such as epigenome-modifying drugs or site-directed epigenetic editing (see Box 2), to potentially provide biological human enhancements.

Bioethicist Allen Buchanan defines a biological enhancement as ‘a deliberate intervention, applying biomedical science, which aims to improve an existing capacity that most or all normal human beings typically have, or to create a new capacity, by acting directly on the body or brain’, whereas Patrick Lin defines it as ‘efforts which aim to improve performance, appearance, or capability besides what is necessary to achieve, sustain, or restore health’, and Greene and Master point out that there is a ‘current emphasis on optimization as opposed to enhancement’ by the departments of defense, referring to the distinction between ‘permanent gene editing for service members’ (as an enhancement) and a less permanent ‘on/off switch for certain genes’ as optimization.^98^ Epigenetic editing could be an example of such an ‘on/off switch’.

In the military context, the added value that epigenetic enhancements could bring to the military is to enable soldiers to successfully achieve specific military objectives with reduced risk of injury and loss of life.^99^ Even if soldiers have voluntarily relinquished certain rights by joining armed forces, military institutions still have a duty to care for their health and safety as much as possible.^100^ In this context, an epigenetic enhancement giving a protection against chemical weapons or toxic gas may also facilitate the realization of the military’s duty to protect soldiers by either mitigating or preventing dangerous or traumatic effects, also avoiding casualties, even if it does not reduce the risk of being exposed to these weapons. Furthermore, epigenetic editing could be envisioned in a therapeutic manner for soldiers who have acquired a condition or disability through service. The purpose of such an intervention would be to use epigenetic technology to correct the disorder, thus restoring the soldier’s performance to a normal level.^101^

However, if providing epigenetic enhancements (eg resistance to the effects of tear gas) to only some soldiers results in a situation where only some benefit from an acquired biological advantage, this might be interpreted as a lack of equity from military institutions and lead to dissent. In discussing military enhancement using gene editing technologies, Greene and Master point out that under the dissent rules, service members may refuse deployment claiming they have not been provided with the same level of protection as others.^102^ Adding to this more general concern formulated in the debate on enhancing members of the military that has not yet specifically considered epigenetic enhancements, Greene and Master focus on genetic enhancement and argue that even if an entire unit received a particular enhancement, future temporary or permanent changes in duty assignment of some soldiers may re-create the inequality of not providing beneficial genetic enhancements to all unit members, thus impacting the moral of troops and the success of the mission.^103^ This concern can likewise be applied to epigenetic enhancements. Unequal access to epigenetic editing for enhancement in the military would lead to a (preventable) lack of uniform protection among soldiers from risks and, thereby, cause an injustice, which would be hard to justify against the duty to reduce risks for all soldiers putting their life on the line. Therefore, equal access to safe, useful, epigenetic editing interventions among soldiers facing comparable levels of risk may need to be ensured to prevent injuries and to promote equity and beneficence.

Although such feats would be considered pre-emptive applications of highly experimental procedures, militaries worldwide have already expressed an interest in developing biological enhancements for their soldiers.^104^ In the following sections, we discuss three more concerns related to militaries having soldiers undergo such enhancements: scientific uncertainty of the target genes and effects, ethical and scientific issues associated with unknown risks, and the ethical and societal issues of dual use of epigenetic editing.

### IV.B. Uncertainty of Target Genes and the Causational Power of Epigenetics

Planning for an epigenetic editing intervention to restore health or improve a trait necessitates identifying the adequate genomic target or targets for a change of epigenetic state that can translate into a biological effect (phenotype). In view of the difficulties associated with this research, the possibility of epigenetic editing for safe and useful military enhancements should not be overstated. Importantly, a phenotype is usually not caused by a single genetic or epigenetic alteration. If for the desired phenotype, epigenetic restoration of function or an enhancement is envisioned, the risk–benefit ratio of the intervention will have to be considered in view of potential secondary phenotypes.^105^ For example, biological approaches to improve ‘physiological fitness’ as one form of enhancement may target various processes, including but not limited to cardiovascular health, cellular and organismal respiration, metabolism, tissue and muscle activity, and nutrition. In this case, the desired biological effect might require an epigenetic approach that simultaneously modulates the expression of a series of genes in specific tissues. This result could also be achieved by modulating the expression of a central gene controlling all aspects of the phenotype (eg many aspects of fitness are associated with angiotensin-converting enzyme (ACE) gene expression).^106^ However, the sheer complexity of epigenetic mechanisms and of gene networks should prompt researchers to consider the increased risk of cascade effects, which may result in undesired or unpredictable effects.^107^ This could hinder the development of epigenetic technologies to achieve a safe human enhancement.

Moreover, there is disagreement within the scientific community as to whether epigenetic signatures or envisioned targeted epigenetic modifications of soldiers should be considered correlated or causal to the phenotype of interest.^108^ For example, if epigenetic signatures are correlational, then it means these signatures only reflect the biological state of the body in an associative manner like a biological fingerprint, but they themselves are not the reason for the phenotype seen. If epigenetic signatures are causal, then it means the specific epigenetic marker is contributing to a phenotype (ie distinct methylation patterns of genomic regions involved in the HPA axis for PTSD is conducive to individuals who manifest PTSD). Therefore, the choice of gene targets in an epigenetic editing intervention will depend upon the identification of epigenetic marks that will induce the desired biological effect. Epigenetic research has been used to characterize the causal and biological impact of an epigenetic signature.^109^ For instance, recent studies using targeted epigenetic editing approaches in cellular cultures and animal models have shown that epigenetic editing mediated histone acetylation, or DNA methylation, of specific genes resulted in the anticipated biological effect.^110^ Beyond demonstrating the feasibility of targeted epigenetic editing technologies, these successes provide first evidence that edited epigenetic signatures can cause biological effects in animals and human cells. Such demonstration of causality is a prerequisite prior to facilitating the design of future epigenetic editing interventions in humans.^111^

### IV.C. Risks Associated with Epigenetic Editing

The dynamic nature of epigenetic regulation, with its vast network of possible target genes, and the high possibility that site-specific epigenetic editing technologies may lead to off-target detrimental effects, make targeted and permanent epigenetic human enhancement technologies more challenging to achieve than theoretically envisioned.^112^ An argument can be made that soldiers would benefit from biological enhancements to keep up with the challenges of modern warfare, appeals to epigenetic editing technology to correct a medical condition should not automatically replace existing avenues of prevention, treatment, and protection against psychological and physical disorders. For instance, psychotherapy can result in substantial positive changes in PTSD symptoms and possibly associated epigenetic marks, and be considered a more justifiable approach than using therapeutic epigenetic editing interventions that come with risks or epi-drug treatment known to cause non-specific epigenetic modifications that alter the plasticity of the brain.^113^ Furthermore, similarly to heritable genome editing, epigenetic-based human editing that would affect the germline/germ cells should only be sought out when medical experts and regulatory bodies have established a clear consensus on the safety and efficacy of such interventions, the proportionality of their use, and their added value as compared to existing, possibly less invasive alternatives.^114^

Accountability for the consequences of cognitive and physical epigenetic enhancements—both on and off the battlefield—must also be considered and delineated. For example, cognitive and physical enhancements may cause soldiers to make riskier decisions on the battlefield or put themselves in life-threatening conditions along with other troops due to self-perceived resilience.^115^ The same enhancements may persist in soldiers after military discharge and prompt them to make riskier everyday decisions in non-combatant and civilian settings, thus potentially undermining their capacity to act as autonomous agents.^116^ These considerations also raise the question of incurred risks in the military and civilian contexts associated with the opportunity to provide soldiers either therapeutic interventions aiming at correcting a specific pathology or enhancing specific cognitive and physical functions above normal human capacity.^117^ In this scenario, unless the possibility of reversing such epigenetic enhancements exists, non-therapeutic epigenetic enhancements may have long-term effects and personality-changing potential that could be deemed riskier than potential therapeutic applications of epigenetic editing aiming to correct a defect.^118^ When assessing the risk of acquiring a specific physical or cognitive capacity, consideration can be given to distinguishing between natural versus artificial enhancements. A therapeutic epigenetic intervention to restore a biological effect that was lost (ie natural enhancement) can be considered less risky than an intervention aiming to generate a biological effect beyond the ‘natural’ limits of human functioning or never seen in a human being (ie an artificial enhancement).^119^ Such artificial enhancements may call for caution as their effects on human health have never been studied. Moreover, equity issues may also materialize when soldiers want to return to civilian life but carry a physiological (or psychological) enhancement that has not been or cannot be removed.^120^ For instance, enhanced soldiers returning to civilian life are still partly militarized and can use physical or mental advantages in inappropriate situations and fail to reappropriate their civilian self.^121^ Since it is currently unclear whether epigenetic editing in humans will become feasible, the effect of stable enhancements on soldiers’ moral responsibility may seem speculative at this point. However, the outcomes of two new clinical trials may require military authorities to further consider the accountability challenges.^122^

In addition to these scientific and ethical challenges for the permissible use of epigenetic editing in the military, Greene and Master point to another issue: they call attention to the risk that under the FDA’s stance on expedited development of DoD medical products, genetic military enhancements based on CRISPR might be used hastily in specific events such as a sudden outbreak of war, before they have been approved.^123^ These uses would not fall under US regulations of clinical trials. They suggest a cautionary approach and smaller scale testing in a controlled research environment permitting the careful evaluation of risks prior to large-scale use and application during combat missions.^124^ The same is to be recommended for epigenetic enhancements in the military, which should not be offered until proven efficient and safe.

### IV.D. Dual Use in the Military and Impact on the Acceptance of Therapeutic Uses

The risk–benefit assessment for military epigenetic enhancement differs from the application of the same technology in other cases, such as sports.^125^ Part of the difference is that the value associated with a reduced risk of death within an armed conflict likely outweighs the value of generating a (potentially unfair) advantage in sports.^126^ However, this difference might diminish when epigenetic editing for military enhancement is misused to create something like an unfair advantage in warfare, which we understand as fostering asymmetrical warfare. If an epigenetic enhancement is to be considered for military use, its above-described risks must, thus, be weighed against the potential benefits of mitigating post-exposure suffering, or death, and against the risk of dual use.^127^

Dual use in research has been defined by the National Academies of Sciences, Engineering, and Medicine as life sciences research that, based on current understanding, can reasonably be anticipated to provide knowledge, information, products, or technologies that could be directly misapplied to pose a significant threat with broad potential consequences to public health and safety, agricultural crops and other plants, animals, the environment, material, or national security.^128^ Evans and Moreno note that dual use, previously referred to as ‘the interplay and transfer of the research, funding, testing, and implementation of technologies across the civilian-military divide, poses distinct socio-ethical issues’.^129^ Moreno and Dando, however, argue the concept had shifted toward the distinction between benign versus malign applications in 2001, when researchers in Australia unintentionally created a mousepox virus that possessed 100 per cent lethality.^130^

With regards to epigenetic editing, such malign use could occur in the military if research from one army into enhancements to protect soldiers against certain substances or weapons prohibited by international law (eg toxic or lethal chemical weapons) is accompanied by the simultaneous development of the same weapons to be used in warfare against the opposing army.^131^ In this case, epigenetic enhancement would no longer be considered solely a preventive measure to protect soldiers against substances used by opposing forces. Alta Charo has described such a scenario as an example of the dual use problem in the context of developing genomic editing technologies.^132^ It is, however, important to note that the purpose of preventing harm toward one’s own soldiers should be weighed against the capacity of inflicting more harm on the opposing party, which even if it needs not be ethically off-limits (depending on the exact form it takes for the search of a competitive advantage), nevertheless may not be subject to the same ethical imperative (eg becoming a malign application of the same technology). Military enhancement by national departments of defense has been envisioned as a reaction to war increasingly becoming asymmetrical with the emergence of guerilla and insurgent forces using chemical and other biological weapons.^133^ Overall, there is a risk that the use of chemical weapons in warfare will increase if epigenetic enhancement to protect soldiers from the side effects of these weapons becomes possible and eventually generalized among armed forces.^134^

While these dual use concerns in epigenetic editing are currently a futuristic scenario, the potential that a country, or an insurgent force, might misuse epigenetic editing to intensify the threat of, for example, chemical weapons, is an important consideration for several reasons. Firstly, legal and ethical safeguards need to be proactively considered to counter this possible future scenario. Secondly, this potential for dual use of epigenetic enhancements in the military context might impact society’s acceptance of research into enhancing and non-enhancing therapeutic applications of epigenetic editing within and outside the military.^135^

## V. CONSIDERATIONS FOR A NORMATIVE FRAMEWORK TO DEFUSE THE ETHICAL MINEFIELD OF EPIGENETICS IN MILITARY, DEFENSE, AND SECURITY CONTEXTS

Following the previous ethical, legal, societal, and scientific evaluations of epigenetic clocks, profiling, and editing, we are, in this final section (prior to a brief conclusion), summarizing some considerations for an ethical framework that can address the translation of epigenetic technologies from research to military, defense, and security applications. A central concern to account for is the tension between respect for the autonomy of soldiers participating in research and their duty to respect the chain of command, which may compromise the informed consent process. Thus, the only acceptable way to engage soldiers in epigenetic research is to ensure that they are not coerced into participating and to minimize adverse consequences for soldiers’ career trajectories.^136^ In this context, the validity of the consent process of soldiers participating in research can be ensured if (i) consent is obtained through an independent party that has no relationship to superiors in the chain of command so that consent or dissent has no adverse consequences for the soldier’s employment status; and (ii) the decision about whether or not, how, when, and to whom to disclose their personal epigenetic-related medical information is reviewed and guided by an independent ethics.^137^

Another central concern is data privacy. There is a need to protect the privacy of sensitive personal health information derived from epigenetic research, as it might shed light not only on a soldier’s risk of developing a disease or reveal previous exposures derived from the military context (eg trauma, toxic gas) but also exposures from before joining the military (eg drug use, smoking). In the latter scenario, candidates who want to join the army might be accepted/enrolled due to their health conditions, a presumption doubly burdensome for those whose illnesses arise from existing socioeconomic inequalities.^138^ Sharing of soldiers’ epigenetic research data to superiors is undesirable, especially if the clinical validity of this information is not well established or if participating soldiers do not want to know this information. In the military research context, we suggest adopting privacy policies prohibiting access to epigenetic information about research participants by hierarchical superiors and establishing strict sanctions in case of privacy breaches. In addition, jurisdictions in which the scope of genetic non-discrimination policies do not clearly apply to soldiers’ epigenetic results (eg see previous discussion on GINA and GNDA) should consider adopting specific epigenetic non-discrimination guidelines to protect soldiers against this new kind of risk of discrimination.

A third concern is the lack of robust evidence for validating the benefits and risks of epigenetic clock applications to determine the chronological age of child soldiers or their exposure to environmental traumas (biological age clock), which will require more research focused on accuracy. The lack of accuracy is illustrated by the current margins of error of 2.7 to 3.6 years, rendering epigenetic age clocks unreliable for estimating the chronological age of child soldiers near 18 years: the risk of wrongfully categorizing a child soldier as an adult would entail the loss of human rights protection provided to children by international conventions.^139^ Before serving as an efficient method for the age determination of paperless child soldiers, the acceptable margin of error of epigenetic age clocks should be limited to within a few months. Moreover, in case of children whose age assessment determines they are over 18, but within the margin of error of the epigenetic age clock, the epigenetic results should either be discarded from the age assessment process, nor be interpreted in favor of the individual claiming to be a child. The UN Committee on the Protection of the Rights of All Migrant Workers and Members of Their Families (CMW) recommends that the best interest of the child principle stated in the *Convention on the Right of the Child* should apply during age assessment procedures of children without identity documents.^140^ More particularly, states performing epigenetic clock-mediated age assessments should apply the presumption of the minority during age assessment procedures, and inconclusive results should be interpreted in favor of the individual claiming to be a child.^141^ Furthermore, age assessments should never be conducted on the sole basis of epigenetic age clocks unless the available evidence confirms the results are completely accurate.

In the future, epigenetic editing could provide an opportunity to better protect soldiers against war zone exposures and thus facilitate their meeting the needs of armed forces. However, this opportunity must be contextualized with the ethical issues and governance challenges raised by this technology. We have outlined considerations that we deem essential for the use of somatic epigenetic editing of soldiers in the previous section. For similar ethical reasons pointed out by international scientific and ethics committees, we do not feel it currently would be appropriate to consider germline epigenetic editing for military use.^142^

The scientific development of somatic epigenetic editing technology (targeting non-germline cells) applied to soldiers should (i) follow a cautionary approach and small-scale testing in a controlled research environment permitting the careful evaluation of risks prior to a larger-scale clinical trial to learn about side effects and durability of the desired biological effects and their applicability to combat missions, and (ii) not be offered until proven efficient and safe.^143^ Moreover, any use of epigenetic technology, in addition to the more general considerations mentioned above, should respect the following three principles: (i) if effective targeted modifications are available, they should be offered equally to each soldier to provide them with the necessary capacities to successfully complete their mission with the smallest risk of death and injury; (ii) every available precaution must be taken to ensure that ‘modified’ soldiers remain without physical and psychological consequences in the short term and in the long term, when they return to civilian life (implying that epigenetic enhancements should be transient and reversible); and (iii) if epigenetic modifications are offered to soldiers, decision makers from military institutions must take accountability for any adverse consequences to soldiers resulting from epigenetic modifications.^144^ If these conditions are not met, the use of epigenetic editing technology may not be deemed justifiable.

## VI. CONCLUSION

This manuscript, based on the authors’ scientific and ELSI knowledge of emerging epigenetic technologies, explores the ethical considerations of using epigenetics in military, defense, and security contexts. Based on the current lack of scientific evidence that does not allow for a robust assessment of the scientific validity, clinical utility, predictive power, and ethical issues of this technology, we argue in favor of caution regarding the use of epigenetic testing, editing, and clock technology in the military. If epigenetics is to be used at all in military applications, we highlight the importance of developing robust scientific knowledge and an ethical governance framework to prevent the risks associated with these emerging technologies and the misuse of epigenetic data in the context of the chain of command culture. In the absence of these safeguards, the safety of soldiers participating in epigenetic research may be compromised, and they may experience a lack of privacy of their epigenetic test results and unfair discrimination. The safety and well-being of soldiers participating in epigenetic editing research must be prioritized, as current scientific knowledge remains uncertain about the causal mechanisms of drugs and environmental factors that can trigger epigenetic pathways, the degree of epigenetic penetrance and inheritance associated with specific epigenetic signatures, and the precision, sustainability, and reversibility of epigenetic editing.

Without careful consideration of these ethical, legal, and scientific issues, epigenetic applications in the military could exacerbate inequities through misuses such as epigenetic surveillance and create an additional safety risk for soldiers rather than providing them with an epigenetic ace in the hole.

## Footnotes

1 The workshop aimed to explore the concerns and opinions of relevant interdisciplinary stakeholders about the opportunities provided by available epigenetic technologies, the status of current scientific research in this field, and ethical and legal challenges to be addressed before—if ever—implementing epigenetic testing and editing technologies in defense and security contexts. The workshop gathered 18 experts working in Canada (1), the USA (5), the UK (4), Germany (3), Switzerland (2), The Netherlands (2), and Taiwan (1), specializing in law (3), ethics (6), medicine (1), sociology (1), philosophy (2), data science (1), and epigenetics (4). See Gratien Dalpé, Katherine Cheung & Yann Joly, *Opportunities and Challenges of Using Epigenetic Technologies in Defence and Security Contexts*, 59 (2022), https://www.genomicsandpolicy.org/Ressources/Dalpe_et_al_2022_Opportunities_and_challenges_of_using_epigenetic_t.pdf.

2 Adrian Bird, *Perceptions of Epigenetics*, 447 Nature 396 (2007); John M. Greally, *A User’s Guide to the Ambiguous Word ‘Epigenetics,’* 19 Nat Rev Mol Cell Biol 207 (2018).

3 C. Dupras et al., *Researcher Perspectives on Ethics Considerations in Epigenetics: An International Survey*, 14 Clin Epigenetics (2022); Greally, *supra* note 2.

4 Clémence Pinel, Barbara Prainsack & Christopher McKevitt, *Markers as Mediators: A Review and Synthesis of Epigenetics Literature*, 13 BioSocieties 276 (2018); Charles Dupras et al., *Epigenetic Discrimination: Emerging Applications of Epigenetics Pointing to the Limitations of Policies Against Genetic Discrimination*, 9 Front. Genet. (2018), https://www.frontiersin.org/articles/10.3389/fgene.2018.00202/full (last visited Mar. 15, 2019).

5 DARPA, *Epigenetic CHaracterization and Observation (ECHO) Proposers Day (Archived)*, (2018), https://www.darpa.mil/news-events/epigenetic-characterization-and-observation-proposers-day (last visited Apr. 19, 2022).

6 Michael R. Hossack et al., *Adverse Childhood Experience, Genes, and PTSD Risk in Soldiers: A Methylation Study*, 185 Mil Med 377 (2020); Katherine C. Bassil, Bart P. F. Rutten & Dorothee Horstkötter, *Biomarkers for PTSD Susceptibility and Resilience, Ethical Issues*, 10 AJOB Neuroscience 122 (2019); Amy Lehrner & Rachel Yehuda, *Biomarkers of PTSD: Military Applications and Considerations*, 5 Eur J Psychotraumatol10.3402/ejpt.v5.23797 (2014); R. Yehuda et al., *Epigenetic Biomarkers as Predictors and Correlates of Symptom Improvement Following Psychotherapy in Combat Veterans with PTSD*, 4 Front Psychiatry (2013), https://www.scopus.com/inward/record.uri?eid=2-s2.0-84885344455&doi=10.3389%2ffpsyt.2013.00118&partnerID=40&md5=a8c4ce4bafa95045179341ae8308853e.

7 Ruoting Yang et al., *Epigenetic Biotypes of Post-Traumatic Stress Disorder in War-Zone Exposed Veteran and Active Duty Males*, 26 Mol Psychiatry 4300 (2021).

8 Valerio Dell’Oste et al., *Metabolic and Inflammatory Response in Post-Traumatic Stress Disorder (PTSD): A Systematic Review on Peripheral Neuroimmune Biomarkers*, 20 Int J Environ Res Public Health 2937 (2023).

9 Christiaan H. Vinkers et al., *Successful Treatment of Post-Traumatic Stress Disorder Reverses DNA Methylation Marks*, 26 Mol Psychiatry 1264 (2021).

10 Robert F. Hillary et al., *An Epigenetic Predictor of Death Captures Multi-Modal Measures of Brain Health*, 26 Mol Psychiatry 3806 (2021).

11
*Id.*

12 Christopher G. Bell et al., *DNA Methylation Aging Clocks: Challenges and Recommendations*, 20 Genome Biol 249 (2019); Hillary et al., *supra* note 10.

13 Bell et al., *supra* note 12.

14 Hillary et al., *supra* note 10.

15 Qian Zhang et al., *Improved Precision of Epigenetic Clock Estimates across Tissues and Its Implication for Biological Ageing*, 11 Genome Med 54 (2019); Steve Horvath et al., *Epigenetic Clock for Skin and Blood Cells Applied to Hutchinson Gilford Progeria Syndrome and Ex Vivo Studies*, 10 Aging 1758 (2018); Alison Abbott, *Can Epigenetics Help Verify the Age Claims of Refugees?*, 561 Nature 15 (2018).

16 Jing Wang & Wen-Hao Zhou, *Epigenetic Clocks in the Pediatric Population: When and Why They Tick?*, 134 Chin Med J (Engl) 2901 (2021); Andrew J. Simpkin et al., *Prenatal and Early Life Influences on Epigenetic Age in Children: A Study of Mother-Offspring Pairs from Two Cohort Studies*, 25 Hum Mol Genet 191 (2016); Rosa H. Mulder et al., *Epigenome-Wide Change and Variation in DNA Methylation in Childhood: Trajectories from Birth to Late Adolescence*, 30 Hum Mol Genet 119 (2021); Teresia Kling, Anna Wenger & Helena Carén, *DNA Methylation-Based Age Estimation in Pediatric Healthy Tissues and Brain Tumors*, 12 Aging (Albany NY) 21037 (2020).

17 Steve Horvath et al., *An Epigenetic Clock Analysis of Race/Ethnicity, Sex, and Coronary Heart Disease*, 17 Genome Biol 171 (2016); Abbott, *supra* note 15; Bell et al., *supra* note 12; Faten Taki & Inmaculada de Melo-Martin, *Conducting Epigenetics Research with Refugees and Asylum Seekers: Attending to the Ethical Challenges*, 13 Clin Epigenetics 105 (2021); Anne-Marie Galow & Shahaf Peleg, *How to Slow down the Ticking Clock: Age-Associated Epigenetic Alterations and Related Interventions to Extend Life Span*, 11 Cells 468 (2022).

18 Jacob H. Goell & Isaac B. Hilton, *CRISPR/Cas-Based Epigenome Editing: Advances, Applications, and Clinical Utility*, 39 Trends Biotechnol 678 (2021); Fabian M. Cortés-Mancera et al., *Gene-Targeted DNA Methylation: Towards Long-Lasting Reprogramming of Gene Expression?*, 1389 *in* DNA Methyltransferases - Role and Function 515 (Albert Jeltsch & Renata Z. Jurkowska eds., 2022), https://link.springer.com/10.1007/978-3-031-11454-0_18 (last visited Jan 23, 2023); Jocelyn Kaiser, *A Gentler Way to Tweak Genes: Epigenome Editing*, 376 Science 1034 (2022); Laura DeFrancesco, *Chroma Medicine and Tune Therapeutics: Two Companies Take up Epigenome Editing*, Nat Biotechnol (2022), https://www.nature.com/articles/d41587-022-00009-x (last visited Sep. 25, 2023); Blythe Sather, *Epigenetic Editing: The Next Generation of Genetic Medicine*, Drug Target Rev (2023), https://www.drugtargetreview.com/article/110654/epigenetic-editing-the-next-generation-of-genetic-medicine/ (last visited Sep. 25, 2023).

19 Samantha D. Creighton et al., *Epigenetic Mechanisms of Learning and Memory: Implications for Aging*, 21 Int J Mol Sci 6918 (2020).

20 A. Florido et al., *Sex Differences in Fear Memory Consolidation via Tac2 Signaling in Mice*, 12 Nat Commun 2496 (2021); Ajinkya S. Sase et al., *Sex-Specific Regulation of Fear Memory by Targeted Epigenetic Editing of Cdk5*, 85 Biol Psychiatry 623 (2019); Creighton et al., *supra* note 19; Florido et al.; Sase et al.

21 Karla Alex & Eva C. Winkler, *Comparative Ethical Evaluation of Epigenome Editing and Genome Editing in Medicine: First Steps and Future Directions*, J Med Ethics (2023), https://jme.bmj.com/content/early/2023/08/01/jme-2022-108888 (last visited Sep. 25, 2023).

22 Cortés-Mancera et al., *supra* note 18.

23 Muneaki Nakamura et al., *CRISPR Technologies for Precise Epigenome Editing*, 23 Nat Cell Biol 11 (2021); Ildefonso I. Rodriguez-Rivera et al., *A Phase 1/2 Open-Label Study to Evaluate the Safety, Tolerability, Pharmacokinetics, Pharmacodynamics, and Preliminary Antitumor Activity of OTX-2002 as a Single Agent and in Combination with Standard of Care in Patients with Hepatocellular Carcinoma and Other Solid Tumor Types Known for Association with the MYC Oncogene (MYCHELANGELO I).*, J Clin Oncol (2023), https://ascopubs.org/doi/pdf/10.1200/JCO.2023.41.4_suppl.TPS627?role=tab (last visited Sep. 28, 2023).

24 Epigenetics, National Institute of Environmental Health Sciences, https://www.niehs.nih.gov/health/topics/science/epigenetics/index.cfm (last visited Sep. 25, 2023).

25 {Citation}.

26 Courtney A. Miller, Susan L. Campbell & J. David Sweatt, *DNA Methylation and Histone Acetylation Work in Concert to Regulate Memory Formation and Synaptic Plasticity*, 89 Neurobiol Learn Mem 599 (2008); Stefan H. Stricker, Anna Köferle & Stephan Beck, *From Profiles to Function in Epigenomics*, 18 Nat Rev Genet 51 (2017).

27 James F. Castellano et al., *Age-Related Memory Impairment Is Associated with Disrupted Multivariate Epigenetic Coordination in the Hippocampus*, 7 PLoS One e33249 (2012).

28 Dupras et al., *supra* note 3.

29 D. Goubert et al., *Epigenetic Editing: Towards Realization of the Curable Genome Concept*, 3 Converg. Sci. Phys. Oncol. 013006 (2017).

30 Marloes L. de Groote, Pernette J. Verschure & Marianne G. Rots, *Epigenetic Editing: Targeted Rewriting of Epigenetic Marks to Modulate Expression of Selected Target Genes*, 40 Nucleic Acids Res 10596 (2012).

31 David Cano-Rodriguez et al., *Writing of H3K4Me3 Overcomes Epigenetic Silencing in a Sustained but Context-Dependent Manner*, 7 Nat Commun 12284 (2016).

32 David Cano-Rodriguez & Marianne G. Rots, *Epigenetic Editing: On the Verge of Reprogramming Gene Expression at Will*, 4 Curr Genet Med Rep 170 (2016); Nakamura et al., *supra* note 23.

33 Charis L. Himeda, Takako I. Jones & Peter L. Jones, *Targeted Epigenetic Repression by CRISPR/dSaCas9 Suppresses Pathogenic DUX4-Fl Expression in FSHD*, 20 Mol Ther Methods Clin Dev 298 (2021); Mariana Brütt Pacheco et al., *Epigenetic Editing in Prostate Cancer: Challenges and Opportunities*, Epigenetics 1 (2021); Philipp Voigt & Danny Reinberg, *Epigenome Editing*, 31 Nat Biotechnol 1097 (2013); Yuta Takahashi et al., *Transgenerational Inheritance of Acquired Epigenetic Signatures at CpG Islands in Mice*, 186 Cell 715 (2023).

34 Cano-Rodriguez and Rots, *supra* note 32.

35 Tim Lewens, *Blurring the Germline: Genome Editing and Transgenerational Epigenetic Inheritance*, 34 Bioethics 7 (2020); Ali Jawaid, Martin Roszkowski & Isabelle M. Mansuy, *Chapter Twelve - Transgenerational Epigenetics of Traumatic Stress*, in 158 Progress in Molecular Biology and Translational Science 273 (Bart P. F. Rutten ed., 2018), https://www.sciencedirect.com/science/article/pii/S187711731830053X (last visited June 13, 2022); Takahashi et al., *supra* note 33.

36 Dell’Oste et al., *supra* note 8.

37 Jawaid, Roszkowski, and Mansuy, *supra* note 35.

38 Raul Artal & Sheldon Rubenfeld, *Ethical Issues in Research*, 43 Best Pract Res Clin Obstet Gynaecol 107 (2017); Miran Epstein, *‘Tell Us What You Want to Do, and We’ll Tell You How to Do It Ethically’—Academic Bioethics: Routinely Ideological and Occasionally Corrupt*, 8 Am J Bioeth 63 (2008); FIN, World Medical Association, *Declaration of Helsinki—Ethical Principles for Medical Research Involving Human Subjects*, World Medical Association (2013), https://www.wma.net/policies-post/wma-declaration-of-helsinki-ethical-principles-for-medical-research-involving-human-subjects/; United Nations Educational Scientific and Cultural Organization, *Universal Declaration on Bioethics and Human Rights*, United Nations Educational Scientific and Cultural Organization (2005), http://portal.unesco.org/en/ev.php-URL_ID=31058&URL_DO=DO_TOPIC&URL_SECTION=201.html;Council for International Organizations of Medical Sciences (CIOMS), *International Ethical Guidelines for Health-Related Research Involving Humans*, (2016), https://cioms.ch/publications/product/international-ethical-guidelines-for-health-related-research-involving-humans/ (last visited Sep 26, 2023).

39 M. J. S. Beauvais, B. M. Knoppers & C. Boscarino, *Navigating the Ethical Maze of Genomics in Canada’s Military*, BMJ Mil Health e001954 (2021); Maxwell J Mehlman, *Bioethics and Soldier Bio-Enhancement*, *in*Transhumanizing War: Performance Enhancement and the Implications for Policy, Society, and the Soldier (H. Christian Breede, Stéphanie A. H. Bélanger, & Stéfanie von Hlatky eds., 2020); Jean-François Caron, A Theory of the Super Soldier: The Morality of Capacity-Increasing Technologies in the Military (2018).

40 Sara Greco, *Conclusion: The Road Ahead*, *in*Transhumanizing War: Performance Enhancement and the Implications for Policy, Society, and the Soldier (H. Christian Breede, Stéphanie A. H. Bélanger, & Stéfanie von Hlatky eds., 2020).

41 Patrick Lin et al., *Super Soldiers (Part 2): The Ethical, Legal, and Operational Implications*, *in*Global Issues and Ethical Considerations in Human Enhancement Technologies: (Steven John Thompson ed., 2014), http://services.igi-global.com/resolvedoi/resolve.aspx?doi=10.4018/978-1-4666-6010-6 (last visited Mar. 14, 2022); Jai Galliott & Mianna Lotz, Super Soldiers: The Ethical, Legal and Social Implications (2015).

42 Interagency Advisory Panel on Research Ethics Government of Canada, *Scope: Interagency Advisory Panel on Research Ethics*, (2016), https://ethics.gc.ca/eng/policy-politique_interpretations_scope-portee.html (last visited Oct. 26, 2022).

43
*Id.*

44 Interagency Advisory Panel on Research Ethics, *TCPS 2*, Tri-Council Policy Statement: Ethical Conduct for Research Involving Humans – TCPS 2 (2018) (2020), http://pre.ethics.gc.ca/eng/policy-politique_tcps2-eptc2_2018.html (last visited Sep. 24, 2019), art. 3.1.

45
Government of Canada, *Research Involving Human Subjects—Approval Procedures*, (1998), https://www.canada.ca/en/department-national-defence/corporate/policies-standards/defence-administrative-orders-directives/5000-series/5061/5061-1-research-involving-human-subjects-approval-procedures.html.

46
Protection of Human Subjects and Adherence to Ethical Standards in Dod-Conducted and Supported Research, (2020), https://www.esd.whs.mil/Portals/54/Documents/DD/issuances/dodi/321602p.pdf; U.S., *Protection of Human Subjects*, Title 32, Code of Federal Regulations, Part 219 (2023), https://www.ecfr.gov/current/title-32/subtitle-A/chapter-I/subchapter-M/part-219.

47 AHLA, *Informed Consent in Military Medical Research*, 8 (2016), https://download.militaryonesource.mil/12038/MOS/AHLA/Informed_Consent_in_Military_Medical_Research.pdf.

48
Protection of Human Subjects and Adherence to Ethical Standards in Dod-Conducted and Supported Research, *supra* note 46.

49
*Id.*

50 GOV.UK, *Governance of Research Involving Human Participants—Part 1: Directive*, 44 (2021), https://assets.publishing.service.gov.uk/government/uploads/system/uploads/attachment_data/file/991593/20210525-JSP536_Part_1_Governance_Research_Human_v3.3_May_21.pdf.

51 Michael L. Gross, *Military Medical Research in Britain and the USA: The Challenge of Informed Consent*, 165 BMJ Mil Health 298 (2019).

52 EU, *Regulation (EU) No 536/2014 of the European Parliament and of the Council of 16 April 2014 on Clinical Trials on Medicinal Products for Human Use, and Repealing Directive 2001/20/EC*, L158/1 (2022), http://data.europa.eu/eli/reg/2014/536/oj/eng (last visited Dec. 2, 2022), art. 28, 1(h).

53 Council of Europe, *Additional Protocol to the Convention on Human Rights and Biomedicine, Concerning Biomedical Research (CETS No. 195)*, (2007), https://rm.coe.int/168008371a (last visited Jul. 13, 2022), art. 12.

54
Steering Committee on Bioethics (CDBI), *Draft Explanatory Report to the Draft Additional Protocol to the Convention on Human Rights and Biomedicine, on Biomedical Research*, (2001) art. 15 (67).

55 Interagency Advisory Panel on Research Ethics, *supra* note 44 at 22, 198.

56 Matthew Beard, Gai Galliott & Sandra Lynch, *Soldier Enhancement: Ethical Risks and Opportunities*, 13 Australian Army Journal 5 (2016); Mehlman, *supra* note 39; Maxwell J. Mehlman & Tracy Yeheng Li, *Ethical, Legal, Social, and Policy Issues in the Use of Genomic Technology by the U.S. Military*, 1 J Law Biosci 244 (2014).

57
Protection of Human Subjects and Adherence to Ethical standards in Dod-Conducted and Supported Research, *supra* note 46.

58 Lin et al., *supra* note 41.

59
World Health Organization, Human Genome Editing: A Framework for Governance (2021), https://apps.who.int/iris/handle/10665/342484.

60 Lin et al., *supra* note 41.

61 Patrick Lin et al., *Super Soldiers (Part 1): What Is Military Human Enhancement?*, *in*Global Issues and Ethical Considerations in Human Enhancement Technologies: (Steven John Thompson ed., 2014), http://services.igi-global.com/resolvedoi/resolve.aspx?doi=10.4018/978-1-4666-6010-6 (last visited Mar 14, 2022); Lin et al., *supra* note 41.

62 Charles Dupras, Katie Michelle Saulnier & Yann Joly, *Epigenetics, Ethics, Law and Society: A Multidisciplinary Review of Descriptive, Instrumental, Dialectical and Reflexive Analyses*, 49 Soc Stud Sci 785 (2019); Nikolajs Zeps et al., *Ethics and Regulatory Considerations for the Clinical Translation of Somatic Cell Human Epigenetic Editing*, 16 Stem Cell Rep 1652 (2021).

63 Charles Dupras & Eline M. Bunnik, *Toward a Framework for Assessing Privacy Risks in Multi-Omic Research and Databases*, 21 Am J Bioeth 46 (2021).

64 Mehlman and Li, *supra* note 56; Nicholas G. Evans & Jonathan D. Moreno, *Yesterday’s War; Tomorrow’s Technology: Peer Commentary on ‘Ethical, Legal, Social and Policy Issues in the Use of Genomic Technologies by the US Military,’* 2 J Law Biosci 79 (2015).

65 Lin et al., *supra* note 41; Carolyn Riley Chapman et al., *Genetic Discrimination: Emerging Ethical Challenges in the Context of Advancing Technology*, 7 J Law Biosci lsz016 (2020); Eline M Bunnik, Marjolein Timmers & Ineke LLE Bolt, *Ethical Issues in Research and Development of Epigenome-Wide Technologies*, 13 Genet Epigenet 251665720913253 (2020); Benjamin M. Helm et al., *Military Health Care Dilemmas and Genetic Discrimination: A Family’s Experience with Whole Exome Sequencing*, 5 Narrat Inq Bioeth 179 (2015).

66 Dupras and Bunnik, *supra* note 63; Eva Winkler et al., *On the Release of Raw Genomic Data to Patients and Study Participants*, Forum Marsilius-Kolleg Bd. 17 (2020) (2020).

67 See the map of existing policy approaches to address genetic discrimination around the world; Genetic Discrimination Observatory, *Genetic Discrimination Observatory*, https://gdo.global/en/gdo-map-approaches.

68 Dupras et al., *supra* note 4; Yann Joly et al., *Looking Beyond GINA: Policy Approaches to Address Genetic Discrimination*, 21 Annu Rev Genomics Hum Genet (2020), https://doi.org/10.1146/annurev-genom-111119-011436 (last visited Feb. 13, 2020); Charles Dupras et al., *Potential (Mis)Use of Epigenetic Age Estimators by Private Companies and Public Agencies: Human Rights Law Should Provide Ethical Guidance*, 12 (2019); Charles Dupras, Yann Joly & Emmanuelle Rial-Sebbag, *Human Rights in the Postgenomic Era: Challenges and Opportunities Arising with Epigenetics*, 59 Soc Sci Inf 12 (2020); UNESCO, *International Declaration on Human Genetic Data*, (2003), https://www.unesco.org/en/legal-affairs/international-declaration-human-genetic-data (last visited Nov. 16, 2022).

69 Canada, *Genetic Non-Discrimination Act*, SC 2017, c 3 (2017), https://www.canlii.org/en/ca/laws/astat/sc-2017-c-3/latest/sc-2017-c-3.html (last visited May 30, 2017), art. 3–5.

70
*Id.*, art. 9.

71 Canada, *National Defence Act*, c. N-5 (2022), https://laws.justice.gc.ca/eng/acts/n-5/page-10.html#h-375814 (last visited Jul. 18, 2022), s. 130(1).

72 Canada, *supra* note 69, art. 2.

73 For example, the G2 biotype associated with the severity of PTSD symptoms in veterans, or the biological age prediction conferred by the GrimAge relating to cognitive decline.

74 Jawaid, Roszkowski, and Mansuy, *supra* note 35; Carrie V. Breton et al., *Exploring the Evidence for Epigenetic Regulation of Environmental Influences on Child Health across Generations*, 4 Commun Biol 769 (2021).

75 Canada, Reference Re Genetic Non-Discrimination Act 2020 SCC 17 (2020).

76 US, *Act of 21 May 2008, Pub L No 110–233, 122 Stat 881*, U.S. Government Information, https://www.govinfo.gov/content/pkg/PLAW-110publ233/pdf/PLAW-110publ233.pdf, Title II; National Human Genome Institute, *Executive Order 13145 to Prohibit Discrimination in Federal Employment Based on Genetic Information*, Genome.gov (2000), https://www.govinfo.gov/content/pkg/FR-2000-02-10/pdf/FR-2000-02-10.pdf (last visited July 13, 2022).

77 Title I of GINA amends these laws: ERISA (1974), PHSA (1944), IRC (1986), SSA (1935). However, each does not apply to both (federal) governmental plans and federal employees and consequently, they (including soldiers) are not subject to GINA. As Military departments are part of the Federal government, Title II of GINA does not apply to them as well as it does not include ‘employees subject to the civil service laws of a State government, governmental agency or political subdivision […]’. See U.S. Government, *Genetic Information Nondiscrimination Act*, 42 USC 2000ff (2008), https://www.govinfo.gov/content/pkg/PLAW-110publ233/pdf/PLAW-110publ233.pdf (last visited July 14, 2022) Title I of GINA amends these laws: ERISA (1974), PHSA (1944), IRC (1986), SSA (1935). However, each does not apply to both (federal) governmental plans and federal employees and consequently, they (including soldiers) are not subject to GINA. As Military departments are part of the Federal government, Title II of GINA does not apply to them as well as it does not include ‘employees subject to the civil service laws of a State government, governmental agency or political subdivision […]’. See; U.S. Congress, *Employee Retirement Income Security Act (ERISA) § 4(b)(1), 29 U.S.C. § 1003(b) (2000)*, (1974); U.S. Congress, *Public Health Service Act (PHSA) of 1944, 42 U.S.C. § 300gg–21 (2000)*, (1944); U.S. Congress, *Internal Revenue Code (IRC) of 1986, 27 U.S.C. §§ 5000(b)(1), 5000(d) (2000)*, (1986); U.S. Congress, *Title XVIII of the Social Security Act (SSA) of 1935, 42 U.S.C. § 1395ss(g)(1) (2000)*, (1935); U.S. Congress, *Civil Rights Act (CRA) of 1964, § 701(f), 42 U.S.C. § 2000e(f) (2000)*, (1964); U.S. Congress, *3 U.S.C. § 411(c)(1)(C) (2000)*; Susannah Baruch & Kathy Hudson, *Civilian and Military Genetics: Nondiscrimination Policy in a Post-GINA World*, 83 Am J Hum Genet 435 (2008).

78 For example, GINA defines genetic tests as ‘an analysis of human DNA, RNA, chromosomes, proteins, or metabolites, that detects genotypes, mutations, or chromosomal changes’. See US, *supra* note 108, Title II, s. 201; It has been argued that DNA methylation can be conceptualized as creating a change in the DNA sequence—eg a change from cytosine (C) to methyl-cytosine (M), also referred to as ‘the fifth base’, but it remains unclear whether such a change would be considered as a ‘mutation’ like originally intended in the law, in which case, the law would permit legal protection concerning the discriminatory use of epigenetic information. See Martine Lappé & Hannah Landecker, *How the Genome Got a Life Span*, 34 New Genet Soc 152 (2015). Also see Dupras et al., *supra* note 5; The notion of chromosomal changes in GINA could also apply to epigenetics (see Box 1) but the ‘analysis of proteins or metabolites that does not detect genotypes, mutations, or chromosomal changes’ does not apply under GINA. US, *supra* note 76.

79 Mauricio De Castro et al., *Genomic Medicine in the Military*, 1 NPJ Genom Med 15,008 (2016).

80 UN Children’s Fund (UNICEF), *The Paris Principles. Principles and Guidelines on Children Associated with Armed Forces or Armed Groups*, (2007), https://www.refworld.org/docid/465198442.html.

81 United Nations, *Optional Protocol to the Convention on the Rights of the Child on the Involvement of Children in Armed Conflict*, General Assembly resolution A/RES/54/263 (2000), https://www.ohchr.org/en/instruments-mechanisms/instruments/optional-protocol-convention-rights-child-involvement-children (last visited July 19, 2022); International Criminal Court, *Rome Statute of the International Criminal Court*, (2002), https://www.icc-cpi.int/sites/default/files/RS-Eng.pdf (last visited July 19, 2022), s. 8(2)(vii).

82 International Committee of the Red Cross, *Protocols I and II Additional to the Geneva Conventions*, (1977), https://www.icrc.org/en/doc/resources/documents/misc/additional-protocols-1977.htm (last visited July 19, 2022), s. 45(3).

83 Abbott, *supra* note 15.

84 Noël Cameron, *The European Refugee Crisis and Biological Age—Is It Right to Use Skeletal Maturity as an Estimate of Chronological Age?*, 43 Ann Hum Biol 415 (2016); T. J. Cole, *The Evidential Value of Developmental Age Imaging for Assessing Age of Majority*, 42 Ann Hum Biol 379 (2015); Pål Skage Dahlberg et al., *A Systematic Review of the Agreement between Chronological Age and Skeletal Age Based on the Greulich and Pyle Atlas*, 29 Eur Radiol 2936 (2019); Jayakumar Jayaraman et al., *The French–Canadian Data Set of Demirjian for Dental Age Estimation: A Systematic Review and Meta-Analysis*, 20 J Forensic Leg Med 373 (2013); Helen M. Liversidge, B. Holly Smith & Melissa Maber, *Bias and Accuracy of Age Estimation Using Developing Teeth in 946 Children*, 143 Am J Phys Anthropol 545 (2010); Upper Tribunal (Immigration and Asylum Chamber), ZM and SK, R (on the Application of) v The London Borough of Croydon (Dental Age Assessment) [2016] UKUT 559 (IAC) (2016), https://www.bailii.org/uk/cases/UKUT/IAC/2016/559.html#_ftn17%20 (last visited July 6, 2022).

85 Abbott, *supra* note 15.

86
*Id.*; Ran Duan et al., *Epigenetic Clock: A Promising Biomarker and Practical Tool in Aging*, 81 Ageing Res Rev 101743 (2022); Also review by Daniel J. Simpson & Tamir Chandra, *Epigenetic Age Prediction*, 20 Aging Cell e13452 (2021).

87 Simpson and Chandra, *supra* note 86.

88 Lisa M. McEwen et al., *The PedBE Clock Accurately Estimates DNA Methylation Age in Pediatric Buccal Cells*, 117 Proc Natl Acad Sci U S A 23329 (2020).

89 UNICEF, *Children Recruited by Armed Forces or Armed Groups*, Child Protection (2021), https://www.unicef.org/protection/children-recruited-by-armed-forces (last visited July 19, 2022).

90 Horvath et al., *supra* note 17.

91 Bell et al., *supra* note 12.

92 Epigenetic clocks are defined by specific panels of DNA methylated markers, See *Id.*

93 Simone Ecker & Stephan Beck, *The Epigenetic Clock: A Molecular Crystal Ball for Human Aging?*, 11 Aging 833 (2019); Zhang et al., *supra* note 15.

94 Taki and de Melo-Martin, *supra* note 17.

95 Marsha Greene & Zubin Master, *Ethical Issues of Using CRISPR Technologies for Research on Military Enhancement*, 15 J Bioeth Inq 327 (2018).

96 Mehlman and Li, *supra* note 56.

97 JASON, The Mitre Corporation, *The $100 Genome: Implications for the DoD*, (2014), http://www.fas.org/irp/agency/dod/jason/hundred.pdf.

98 Allen Buchanan, *The Landscape of the Enhancement Debate*, *in*Beyond Humanity? (2011), https://oxford.universitypressscholarship.com/10.1093/acprof:oso/9780199587810.001.0001/acprof-9780199587810-chapter-1 (last visited July 12, 2022); Greene and Master, *supra* note 95; Lin et al., *supra* note 61.

99 Lin et al., *supra* note 41.

100
Jean-François Caron, A Theory of the Super Soldier: The Morality of Capacity-Increasing Technologies in the Military (2018).

101 Mara Almeida & Rui Diogo, *Human Enhancement: Genetic Engineering and Evolution*, 2019 Evol Med Public Health 183 (2019).

102 Greene and Master, *supra* note 95.

103
*Id.*

104 Dianne Price, *DARPA Grants Arizona State University up to $38.8 Million to Create Epigenetic Tool for Fight against Weapons of Mass Destruction*, ASU Now: Access, Excellence, Impact (2019), https://asunow.asu.edu/20190722-darpa-grants-asu-388-million-create-epigenetic-tool-fight-against-weapons-mass-destruction (last visited Jan. 25, 2021).

105 Almeida and Diogo, *supra* note 101.

106 Adrián Montes-de-Oca-García et al., *Influence of ACE Gene I/D Polymorphism on Cardiometabolic Risk, Maximal Fat Oxidation, Cardiorespiratory Fitness, Diet and Physical Activity in Young Adults*, 18 Int J Environ Res Public Health 3443 (2021).

107 For example, an upstream epigenetic change at a certain gene locus resulting in unforeseen downstream events: the up- or downregulation of one or many downstream genes. Almeida and Diogo, *supra* note 101; de Groote, Verschure, and Rots, *supra* note 30; Aleksandra Majchrzak-Celińska, Anna Warych & Mikołaj Szoszkiewicz, *Novel Approaches to Epigenetic Therapies: From Drug Combinations to Epigenetic Editing*, 12 Genes 208 (2021).

108 Amita Bansal & Rebecca A. Simmons, *Epigenetics and Developmental Origins of Diabetes: Correlation or Causation?*, 315 Am J Physiol Endocrinol Metab E15 (2018); Goell and Hilton, *supra* note 18.

109 Vinkers et al., *supra* note 9.

110 Fernando J. Bustos et al., *Epigenetic Editing of the Dlg4/PSD95 Gene Improves Cognition in Aged and Alzheimer’s Disease Mice*, 140 Brain 3252 (2017); Samrat Roy Choudhury et al., *CRISPR-dCas9 Mediated TET1 Targeting for Selective DNA Demethylation at BRCA1 Promoter*, 7 Oncotarget 46545 (2016); Himeda, Jones, and Jones, *supra* note 33; M. Okada et al., *Stabilization of Foxp3 Expression by CRISPR-dCas9-Based Epigenome Editing in Mouse Primary T Cells*, 10 Epigenet Chromatin (2017); Joseph Yusup Shin et al., *Epigenetic Activation and Memory at a TGFB2 Enhancer in Systemic Sclerosis*, 11 Sci Transl Med eaaw0790 (2019).

111 Sase et al., *supra* note 20; Song-Jun Xu et al., *Chromatin-Mediated Alternative Splicing Regulates Cocaine-Reward Behavior*, 109 Neuron 2943 (2021).

112 Katherine Huerne et al., *Auditing the Editor: A Review of Key Translational Issues in Epigenetic Editing*, 5 CRISPR J 203 (2022).

113 Rachel Yehuda et al., *Epigenetic Biomarkers as Predictors and Correlates of Symptom Improvement Following Psychotherapy in Combat Veterans with PTSD*, 4 Front. Psychiatry (2013), http://journal.frontiersin.org/article/10.3389/fpsyt.2013.00118/abstract (last visited Sep. 1, 2021).

114 Huerne et al., *supra* note 112.

115 Heather A. Harrison Dinniss & Jann K. Kleffner, *Soldier 2.0: Military Human Enhancement and International Law*, *in*Dehumanization of Warfare 163 (Wolff Heintschel von Heinegg, Robert Frau, & Tassilo Singer eds., 2018), http://link.springer.com/10.1007/978-3-319-67266-3_10 (last visited June 8, 2022).

116
*Id.*; Beard, Galliott, and Lynch, *supra* note 56; Bernard Barrera, *The Enhanced Soldier: The Needs and Prospects of Increasing the Fighter’s Abilities*, (2021), https://www.armyupress.army.mil/Journals/Military-Review/Online-Exclusive/2021-French-OLE/part1-The-Enhanced-Soldier/; Arthur Saniotis & Jaliya Kumaratilake, *Amphetamines, Cognitive Enhancement and Their Implications for Medical Military Ethics*, 19 J Mil Ethics 69 (2020); Marcin Orzechowski & Florian Steger, *Promises and Perils of Neuroenhancement and Its Perspectives for Military Ethics*, Acta Universitatis Lodziensis. Folia Philosophica. Ethica-Aesthetica-Practica 11 (2018).

117 Saniotis and Kumaratilake, *supra* note 116.

118 James K. Nuñez et al., *Genome-Wide Programmable Transcriptional Memory by CRISPR-Based Epigenome Editing*, 184 Cell 2503 (2021).

119 Lin et al., *supra* note 61.

120 Beard, Galliott, and Lynch, *supra* note 56; Paul Comeau, *A Framework to Assess the Military Ethics of Human Enhancement Technologies*, 18 (2017), https://cradpdf.drdc-rddc.gc.ca/PDFS/unc279/p805510_A1b.pdf.

121 Evans and Moreno, *supra* note 64.

122 Montpellier University Hospital, *Exploiting Epigenome Editing in Kabuki Syndrome: A New Route Towards Gene Therapy for Rare Genetic Disorders (Epi-KAB)*, ClinicalTrials.gov, https://beta.clinicaltrials.gov/study/NCT03855631?distance=50&term=epigenetic%20crispr&rank=1 (last visited Nov. 3, 2022).

123 Greene and Master, *supra* note 95.

124
*Id.*

125
World Health Organization, *supra* note 59.

126
*Id.*; Zeps et al., *supra* note 62.

127 Greene and Master, *supra* note 95.

128
Board on Life Sciences, Division on Earth and Life Studies, & National Academies of Sciences, Engineering, and Medicine, Governance of Dual Use Research in the Life Sciences: Advancing Global Consensus on Research Oversight: Proceedings of a Workshop (James Revill, Jo Husbands, & Katherine Bowman eds., 2018), https://www.nap.edu/catalog/25154 (last visited Aug. 11, 2022).

129 Evans and Moreno, *supra* note 64; National Research Council, Biotechnology Research in an Age of Terrorism (2004), http://www.nap.edu/catalog/10827 (last visited Nov. 17, 2022).

130 Jonathan Moreno & Malcolm Dando, *The Limits of Dual Use - Responses*, Issues Sci Technol (July 31, 2018), https://issues.org/the-limits-of-dual-use/ (last visited Aug. 11, 2022); Michael J. Selgelid & Lorna Weir, *The Mousepox Experience*, 11 EMBO Rep 18 (2010).

131 R. Alta Charo, *Legal Issues Related to Human Germline Genome Editing in the United States*, *in*Rechtliche Aspekte der Genom-Editierung an der menschlichen Keimbahn : A Comparative Legal Study 439 (Jochen Taupitz & Silvia Deuring eds., 2020), https://doi.org/10.1007/978-3-662-59028-7_21 (last visited Aug. 11, 2022); Board on Life Sciences, Division on Earth and Life Studies, and National Academies of Sciences, Engineering, and Medicine, *supra* note 128.

132 Charo, *supra* note 131; Board on Life Sciences, Division on Earth and Life Studies, and National Academies of Sciences, Engineering, and Medicine, *supra* note 128.

133 Greene and Master, *supra* note 95.

134
Seumas Miller, Dual Use Science and Technology, Ethics and Weapons of Mass Destruction (2018), http://link.springer.com/10.1007/978-3-319-92606-3 (last visited Dec. 5, 2022).

135 Wen-Di Pei et al., *Epigenome Editing by CRISPR/Cas9 in Clinical Settings: Possibilities and Challenges*, 19 Brief Funct Genomics 215 (2020); Emily L. Howell et al., *Enhanced Threat or Therapeutic Benefit? Risk and Benefit Perceptions of Human Gene Editing by Purpose and Heritability of Edits*, 25 J Risk Res 139 (2022).

136 Josep Santaló & María Berdasco, *Ethical Implications of Epigenetics in the Era of Personalized Medicine*, 14 Clin Epigenet 44 (2022); Lehrner and Yehuda, *supra* note 6; Helm et al., *supra* note 65; De Castro et al., *supra* note 79.

137 Interagency Advisory Panel on Research Ethics, *supra* note 46; Protection of Human Subjects and Adherence to Ethical Standards in Dod-Conducted and Supported Research, *supra* note 48; GOV.UK, *supra* note 52; Regulation (EU) No 536/2014 of the European Parliament and of the Council of 16 April 2014 on clinical trials on medicinal products for human use, and repealing Directive 2001/20/EC Text with EEA relevance, *supra* note 54.

138 Yann Joly et al., *Epigenetics ELSI: Darker Than You Think?*, 32 Trends Genet 591 (2016).

139 UN Children’s Fund (UNICEF), *The Paris Principles. Principles and Guidelines on Children Associated with Armed Forces or Armed Groups*, (2007), https://www.refworld.org/docid/465198442.html; United Nations, *supra* note 81, art. 1–2; International Criminal Court, *supra* note 81 at 8(2)(vii); International Committee of the Red Cross, *supra* note 82 at 45(3).

140 United Nations, *Convention on the Rights of the Child*, New York, 20 November 1989 United Nations, Treaty Series, vol. 1577, p. 3 (1989), art. 3(1); UN Committee on the Protection of the Rights of All Migrant Workers and Members of Their Families (CMW), *Joint General Comment No. 3 (2017) of the Committee on the Protection of the Rights of All Migrant Workers and Members of Their Families and No. 22 (2017) of the Committee on the Rights of the Child on the General Principles Regarding the Human Rights of Children in the Context of International Migration*, 32(h) (2017), https://www.refworld.org/docid/5a1293a24.html.

141
John Dorber, *Age Assessment for Children in Migration: A Human Rights-Based Approach*, (2019), https://rm.coe.int/ageassessmentchildrenmigration/168099529f (last visited Dec. 21, 2022); Directive 2011/95/Eu of the European Parliament And of the Council of 13 December 2011 on standards for the qualification of third-country nationals or stateless persons as beneficiaries of international protection, for a uniform status for refugees or for persons eligible for subsidiary protection, and for the content of the protection granted, https://eur-lex.europa.eu/LexUriServ/LexUriServ.do?uri=OJ:L:2011:337:0009:0026:en:PDF#:~:text=The%20purpose%20of%20this%20Directive,content%20of%20the%20protection-granted. (last visited Jan. 3, 2023), art. 5; Statement of Good Practice, D.5.1, D.5.3 (2009), https://www.refworld.org/docid/415450694.html;Guidelines on Policies and Procedures in dealing with Unaccompanied Children Seeking Asylum, 5.11(c) (1997), https://www.unhcr.org/3d4f91cf4.pdf (last visited Jan. 3, 2023); UNHCR Executive Committee of the High Commissioner’s Programme, *Conclusion on Children at Risk No. 107 (LVIII)—2007*, g(ix) (2007), https://www.unhcr.org/excom/exconc/4717625c2/conclusion-children-risk.html (last visited Jan. 3, 2023); UNHCR, *Guidelines on International Protection: Child Asylum Claims under Articles 1(A)2 and 1(F) of the 1951 Convention and/or 1967 Protocol Relating to the Status of Refugees*, 75 (2009), https://www.unhcr.org/50ae46309.pdf (last visited Jan. 3, 2023); Council of Europe Convention on Action against Trafficking in Human Beings, https://rm.coe.int/168008371d (last visited Jan. 3, 2023), art. 10(3); Directive 2011/36/Eu of the European Parliament and of the Council of 5 April 2011 on preventing and combating trafficking in human beings and protecting its victims, and replacing Council Framework Decision 2002/629/JHA, (2011), https://eur-lex.europa.eu/legal-content/EN/TXT/PDF/?uri=CELEX:32011L0036&from=en (last visited Jan. 3, 2023), art. 13(2); Directive 2013/32/EU of the European Parliament and of the Council of 26 June 2013 on common procedures for granting and withdrawing international protection (recast), (2013), https://eur-lex.europa.eu/legal-content/EN/TXT/PDF/?uri=CELEX:32013L0032&from=en (last visited Jan. 3, 2023), art. 25(5); Committee on the Rights of the Child, *Treatment of Unaccompanied and Separated Children Outside Their Country of Origin*, 31(a) (2005), https://www2.ohchr.org/english/bodies/crc/docs/GC6.pdf; Council of Europe Parliamentary Assembly, *Unaccompanied Children in Europe: Issues of Arrival, Stay and Return*, 5.10 (2011), https://assembly.coe.int/nw/xml/XRef/Xref-XML2HTML-en.asp?fileid=17991&lang=EN (last visited Jan. 3, 2023).

142 Almeida and Diogo, *supra* note 101; Carolyn Brokowski & Mazhar Adli, *CRISPR Ethics: Moral Considerations for Applications of a Powerful Tool*, 431 J Mol Biol 88 (2019); Almeida and Diogo, *supra* note 101; Committee on Human Gene Editing: Scientific, Medical, and Ethical Considerations et al., Human Genome Editing: Science, Ethics, and Governance (2017), https://www.nap.edu/catalog/24623 (last visited Aug. 11, 2022).

143 Greene and Master, *supra* note 95.

144
Comité d’Éthique de la Défense, *Avis Portant Sur Le Soldat Augmenté*, (2020), https://www.defense.gouv.fr/sites/default/files/ministere-armees/20200921_Comit%C3%A9%20d%27%C3%A9thique%20de%20la%20d%C3%A9fense%20-%20Avis%20soldat%20augment%C3%A9.pdf (last visited July 13, 2022); Comeau, *supra* note 120.

